# Perioperative blood transfusions and survival in patients with non-small cell lung cancer: a retrospective study

**DOI:** 10.1186/1471-2253-13-42

**Published:** 2013-11-15

**Authors:** Juan P Cata, Varun Chukka, Hao Wang, Lei Feng, Vijaya Gottumukkala, Fernando Martinez, Ara A Vaporciyan

**Affiliations:** 1 Department of Anesthesiology and Perioperative Medicine, The University of Texas - MD Anderson Cancer Center, 1515 Holcombe Blvd, Houston, TX, USA; 2 Department of Biostatistics, The University of Texas - MD Anderson Cancer Center, Houston, TX, USA; 3 Department of Laboratory Medicine, The University of Texas - MD Anderson Cancer Center, Houston, TX, USA; 4 Department of Thoracic and Cardiovascular Surgery, The University of Texas - MD Anderson Cancer Center, Houston, TX, USA

## Abstract

**Background:**

Perioperative blood transfusions have been associated with poor clinical outcomes in the context of oncological surgery. Current literature is inconclusive whether blood transfusions are linked to shorter recurrence free and overall survival after lung cancer surgery. We hypothesize that blood transfusions in patients undergoing surgery for non-small cell lung cancer are associated with poor oncological survival.

**Methods:**

After IRB approval, perioperative data from 636 patients who underwent lung cancer surgery was collected. Patients were evaluated for time to tumor recurrence and overall survival.

**Results:**

60 patients were transfused and 576 subjects were not. Patients who received transfusion were more likely to have more advanced disease (p = 0.018), and preoperative low hemoglobin concentrations (p < 0.0001) compared to non-transfused patients. In the multivariable Cox regression analysis, blood transfusion was associated with a significant reduction in recurrence free survival (p = 0.025), HR: 1.55 (95% CI: 1.06-2.27) and overall survival (p = 0.0002) HR: 2.04 (95% CI: 1.41-2.97). However, analysis after propensity score matching between the two groups revealed that the effect of blood transfusion was significant for reduction in overall survival (p = 0.0356), HR: 1.838 (95% CI: 1.04-3.22) but not for recurrence free survival (p = 0.1460), HR: 1.493 (95% CI: 0.87-2.56).

**Conclusions:**

Perioperative administration of red blood cells appears be associated with a decreased overall survival but not recurrence free survival after lung cancer surgery. Our study has the limitations of a retrospective review. Hence, our results should be confirmed by a prospective randomized control trial.

## Background

Surgery remains the most effective treatment for stage I to IIIa non-small cell lung cancer (NSCLC), which is the second most common cancer in the United States [1]. Although the rate of perioperative blood transfusions in patients with NSCLC is relatively low, the number of patients who will receive packed red blood cells (pRBCs) across the world during surgery for NSCLC is not insignificant and hence their exposition to adverse effects of blood transfusions [2,3]. Unfortunately, administration of pRBCs is associated with a distinct pathology of immunosuppression, known as transfusion related immune suppression (TRIM). TRIM has been linked to a number of complications including effects on cancer recurrence [4]. TRIM is considered to contribute to progression of minimal residual disease (MRD) to clinical metastasis particularly in patients who do not receive postoperative chemo-radiation as adjuvant therapy after surgery [5-9]. Several mechanisms have been described to be responsible of TRIM. The accumulation of tumor growth factors, lysophophatidylcholines and pro-tumoral cytokines in the units of pRBCs along with a predominant immunosuppressive response of the host in reaction to microparticles and fragmented RBCs facilitate the growth of cancer cells [10]. Moreover, patients undergoing lung cancer surgery have significant impairment in their ability to attack cancer cells as the result of cancer itself, surgery, anesthetics and analgesics given intra- and postoperatively [11]. Thus, TRIM may further impair the innate immunity of these patients after surgery.

The results of a Cochrane review indicate that blood transfusions are associated with an increased risk of cancer recurrence in patients with colorectal cancer [12]. Several retrospective studies in patients with NSCLC demonstrated that perioperative blood transfusions were associated with poorer disease-free and overall survival [2,13-15]. However, other studies have concluded to the contrary [16,17]. The published retrospective studies had several limitations including small sample size, differences in the surgical procedures performed, differences in the type of blood products administered, differences in the follow-up duration and the presence of confounding tumor and treatment related factors [14,15,17]. Thus, we decided to investigate whether the administration of pRBCs to patients with stage 1–3 NSCLC who underwent tumor resection at MD Anderson Cancer Center had an effect on their recurrence-free survival and overall survival.

## Methods

### Patient selection

After approval from MD Anderson Cancer Center institutional review board, we recorded information from 636 patients with stage 1-3a NSCLC cancer who underwent lobectomies, sublobar resection, sleeve lobectomy, or pneumonectomy from 2004 through 2006. Patients who received allogenic pRBCs within 30 days before surgery and during their postoperative hospitalization were compared with those who received no transfusion. We collected demographic (age, gender, body mass index and ASA physical status), co-morbidities (smoking, alcohol consumption, diabetes mellitus, coronary artery disease, creatinine higher than 2 mg/dL, stroke, and preopeative anemia defined as hemoglobin lower than 12 g/dL), tumor-related variables (tumor size and stage) and treatment-related variables (preoperative and postoperative chemotherapy and radiation). We also recorded the number of pRBCs units transfused.

### Statistical analysis

The primary endpoint of interest was recurrence free survival (RFS), defined as the time in months from surgery to recurrence or death, whichever event occurred first. Overall survival (OS) was defined as the time in months from surgery to death from any cause. The exposure variable of interest was blood transfusion status (yes or no) for surgery. Other prognostic variables of interest included age at surgery, gender, BMI, ASA, cancer stage, tumor size, smoking status, alcohol consumption, patient comorbidities, preoperative hemoglobin level, preoperative and postoperative chemoradiation.

Propensity score analysis was undertaken in an attempt to adjust for potential bias associated with factors related to the decision to undergo blood transfusion. This statistical methodology has often been used in observational studies to control for nonrandom treatment assignment of patients by adjusting for differences in covariates between the treatment groups. To control for factors that may confound the relationship between blood transfusion and RFS or OS, we determined the propensity score to receive blood transfusion for each patient, using multivariable logistic regression. The covariates included in the analysis were: age at surgery, gender (F or M), BMI, ASA (2, 3, or 4), stage (1, 2, or 3), and preoperative hemoglobin level. Given the propensity scores for all patients, we identified sets of patients, one transfused patient randomly matched with two who did not undergo blood transfusion, using a 5-to-1 digit greedy match algorithm. The differences in propensity scores in each set were no more than 0.06. We used absolute standardized differences to assess balance in the baseline variables between patients who underwent blood transfusion and those who did not undergo blood transfusion in the matched cohort. The absolute standardized differences for all baseline covariates were< 9% in the matched cohort.

For the prematching cohort, differences in demographic, clinical, and tumor characteristics between groups of patients who underwent blood transfusion and those who did not undergo blood transfusion were compared by using Fisher’s exact test or Chi-square test for categorical variables and Wilcoxon rank sum test for continuous variables. Kaplan-Meier method was used to evaluate the effect of patient characteristics on RFS and OS. Multivariable Cox proportional hazards models were used to determine the effect of blood transfusion on RFS and OS after adjusting for the prognostic variables.

To get the matched cohort, 37 patients were excluded due to missing information on RFS, where 9 of them were transfused patients. One of the remaining 51 transfused patients did not have a match and five of the 51 transfused patients did not have a second match in the non-transfused group (Table 1). For the matched cohort (n = 135), differences in demographic, clinical, and tumor characteristics between matched pairs were evaluated using generalized estimating equation method. Cox proportional hazards models stratified on the matched pairs were fitted to determine the effect of blood transfusion on RFS and OS for the matched cohort. Kaplan-Meier curves by transfusion status were generated for the prematching cohort and the matched cohort. All tests were 2-sided. P values < 0.05 were considered statistically significant. All analyses were conducted using SAS (version 9.1; SAS Institute, Cary, NC) and S-plus (version 8.0; TIBCO Software Inc., Palo Alto, California) statistical software.

**Table 1:** Patient and tumor characteristics by blood transfusion status before and after matching

**Factors**	**All patients**		**P value**	**Matched patients**		**P value**
	**Non-transfused**	**Transfused**		**Non-transfused**	**Transfused**	
Total n	576 (90.6%)	60 (9.4%)		90	45	
Age (SD)	65.16 (10.54)	66.21 (9.46)	0.91	65.08 (10.8)	65.8 (10.12)	0.73
BMI (SD)	27.06 (5.31)	26.88 (7.33)	0.15	27.37 (5.76)	27.02 (7.59)	0.79
Gender			0.43			0.80
Female	267 (89.6%)	31 (10.4%)		48 (67.6%)	23 (32.4%)	
Male	309 (91.4%)	29 (8.6%)		42 (65.6%)	22 (34.4%)	
ASA			0.48			0.82
2	62 (93.9%)	4 (6.1%)		9 (75%)	3 (25%)	
3	480 (90.4%)	51 (9.6%)		75 65.8%)	39 (34.2%)	
4	33 (86.8%)	5 (13.2%)		6 (66.7%)	3 (33.3%)	
Stage			0.01			0.92
1	328 (93.4%)	23 (6.6%)		32 (65.3%)	17 (34.7%)	
2	115 (87.8%)	16 (12.2%)		25 (65.8%)	13 (34.2%)	
3	131 (86.2%)	21 (13.8%)		33 (68.8%)	15 (31.3%)	
Hb g/dL, (SD)	13.43 (1.42)	12.08 (1.58)	< 0.0001	12.4 (1.3)	12.3 (1.4)	0.73

A p value < 0.05 was considered statistically significant. *BMI* Body mass index. *ASA* American Society of Anesthesiologist physical status. *Hb* Hemoglobin.

## Results

### Patient characteristics

We used a total of 636 patients in our analysis, 60 in the blood transfusion group, and 576 in the no-tranfusion group. The median follow-up time for the entire study cohort was 5.3 years for the censored observations. The median follow-up time was 5.3 years among patients who received blood transfusions and 5.3 years among patients who did not undergo have blood transfusions. Table 1 lists the characteristics of patients in the blood transfusion and no-transfusion groups before and after propensity score matching.

Prior to matching, patients who received blood transfusions differed from those who did not have transfusion in terms of important prognostic factors. Patients who received transfusion were more likely to have stage II to III disease (p = 0.018) compared to patients who did not undergo transfusion. The transfused patients were also more likely to have low hemoglobin levels (p < 0.0001). Matching using the propensity score substantially reduced the imbalance. None of the variables was significantly different between the transfused patients and non-transfused patients in the matched cohort.

### Recurrence free survival

**Figure 1:**
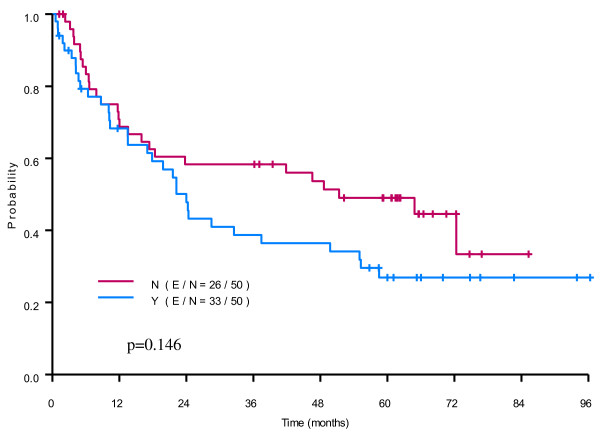
**The Kaplan-Meier curve and p-value from stratified Cox proportional hazards model for RFS are depicted in the figure.** Our analysis showed no association between blood transfusion and recurrence free survival.

Among the 599 out of 636 patients with recurrence information, the median RFS time was of 68.79 (95% confidence interval (CI): 61.4-81.8) months with a RFS rate at 3 and 5 years of 65% (95% CI: 61-69%) and 54% (95% CI: 50-59%). The log-rank test showed that age > 66 or older, BMI 25 or lower, male, high ASA physical status, high cancer stage, ASA physical status, preoperative Hb lower than 12 g/dL, or receiving chemoterapy and radiotherapy were associated with worse RFS (Table 2). Also, the transfused patients had a worse RFS compared to those non-transfused patients (p = 0.0003). In addition, the number of units transfused (0, 1–3, 4–9 and more than 10) was a factor associated with worse RFS (p = 0.001). Tumor size has a significant impact on RFS from unicovariate Cox proportional hazards model (data not shown). In the multivariable Cox regression analysis, blood transfusion was still significantly associated with worse RFS (p = 0.02, harzard ratio (HR): 1.55 (95% CI: 1.06-2.27)) with the adjustment of age, gender, BMI, ASA, cancer stage, and hemoglobin level in the model (Table 3). In the analysis with the matched cohort, the effect of blood transfusion on RFS was not significant (p = 0.14) (Table 3, Figure 1).

**Table 2:** Recurrence free survival (RFS) by patient characteristics

**Variable**	**Level**	**N**	**Event**	**Median RFS time in months (95% CI)**	**RFS rate at 3 years (95% CI)**	**RFS rate at 5 years (95% CI)**	**P-value**
	**All patients**	**599**	**277**	**68.79 (61.4,81.8)**	**0.65 (0.61,0.69)**	**0.54 (0.5,0.59)**	
Age (years)	< 66	277	107	81.7 (74.24,NA)	0.69 (0.64,0.75)	0.61 (0.55,0.67)	0.008
	> = 66	322	170	56.67 (43.69,68.79)	0.61 (0.56,0.67)	0.49 (0.44,0.55)	
BMI	< =25	223	115	53.48 (37.42,NA)	0.58 (0.51,0.65)	0.46 (0.4,0.54)	0.02
	>25	376	162	75.33 (67.05,NA)	0.69 (0.65,0.74)	0.59 (0.54,0.65)	
Gender	F	281	114	87.52 (72.34,NA)	0.71 (0.65,0.76)	0.62 (0.56,0.68)	0.0002
	M	318	163	53.78 (41.85,68.66)	0.6 (0.55,0.66)	0.47 (0.42,0.54)	
ASA	2	63	23	NA (63.24,NA)	0.7 (0.59,0.83)	0.62 (0.5,0.76)	0.04
	3	500	230	69.97 (60.35,NA)	0.65 (0.61,0.69)	0.55 (0.5,0.59)	
	4	35	24	39.13 (20.4,NA)	0.56 (0.42,0.76)	0.38 (0.25,0.59)	
Stage	1	332	121	87.19 (81.8,NA)	0.75 (0.71,0.8)	0.64 (0.59,0.7)	< 0.0001
	2	125	74	48.65 (27.73,66.52)	0.55 (0.46,0.64)	0.43 (0.35,0.53)	
	3	140	82	31.44 (20.01,67.05)	0.49 (0.41,0.58)	0.4 (0.33,0.5)	
Preoperative	N	497	222	74.24 (65.54,NA)	0.67 (0.63,0.72)	0.57 (0.52,0.61)	0.001
Hb < 12 g/dL	Y	101	55	39.13 (23.75,NA)	0.52 (0.43,0.63)	0.42 (0.33,0.54)	
BT	N	548	244	74.24 (66.52,7.52)	0.67 (0.63,0.71)	0.57 (0.52,0.61)	0.0002
	Y	51	33	23.98 (16.98,55.22)	0.4 (0.28,0.57)	0.29 (0.18,0.45)	
Number of units	0	548	244	74.24 (66.52,87.52)	0.67 (0.63 0.71)	0.57 (0.52,0.61)	0.001
Transfused	1-3	31	19	24.34 (10.32,NA)	0.4 (0.25,0.63)	0.32 (0.19,0.55)	
	4-9	12	8	24.24 (22.21,NA)	0.42 (0.2,0.88)	0.21 (0.06,0.72)	
	>10	6	5	17.85 (3.45,NA)	0.33 (0.11,1)	0.17 (0.03,1)	
Preoperative	N	501	217	77.63 (66.69,NA)	0.69 (0.65,0.73)	0.58 (0.53,0.62)	< 0.0001
Chemo-radiation	Y	98	60	21.71 (13.83,55.22)	0.45 (0.36,0.56)	0.38 (0.29,0.49)	
Postoperative	Chemo only	132	57	81.8 (60.35,NA)	0.67 (0.59,0.76)	0.58 (0.5,0.68)	< 0.0001
Therapy	Chemo/Radiation	54	35	34.92 (17.35,74.24)	0.49 (0.36,0.65)	0.39 (0.28,0.56)	
	None	378	160	75.33 (66.52,NA)	0.69 (0.65,0.74)	0.58 (0.53,0.63)	
	Radiation only	35	25	20.66 (3.47,51.35)	0.38 (0.25,0.59)	0.27 (0.15,0.46)	

P-values from the log-rank test (univariable analysis), p < 0.05 is considered statistically significant. *BMI* Body mass index. *ASA* American Society of Anesthesiologist physical status. *Hb* Hemoglobin.

**Table 3:** Association between blood transfusion (Y vs. N) and recurrence free survival

	**p-value**	**HR**	**95% CI**	
Unadjusted (univariate analysis), before matching (E/N = 277/599)	0.0003	1.97	1.37	2.84
Adjusted for covariates (multivariate analysis)* (E/N = 277/595)	0.0253	1.54	1.05	2.27
Propensity-matched, stratifying on the matched pair (E/N = 78/135)	0.14	1.49	0.87	2.56

*Covariate in the final model included age, gender, BMI, ASA, Stage, and Hb.

### Overall survival

**Figure 2:**
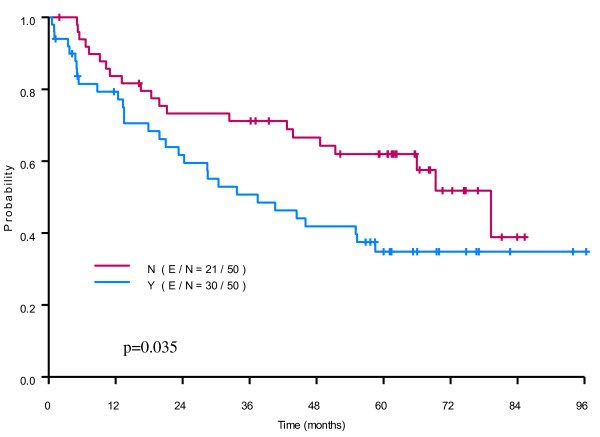
**The Kaplan-Meier curve and p-value from stratified Cox proportional hazards model for OS are shown in the figure.** Our analysis showed a statistically significant association between blood transfusion and overall survival.

The OS rate of all patients was 72% (95% CI: 69-76%) and 62% (95% CI: 58-66%) at 3 and 5 years respectively with a median OS time of 82.39 months (95% CI: 78.65-NA). The univariable analysis demonstrated that blood transfusion was associated with worse overall survival outcomes (Figure 2A). Other prognostic variables that had a significant impact on OS from the log-rank test were age (p < 0.0001), BMI (p = 0.0014), gender (p < 0.0001), ASA score (p = 0.04), cancer stage (p < 0.0001), number of transfused units (p < 0.0001), preoperative and postoperative chemoradiation (< 0.0001) and preoperative Hb (p = 0.001) (Table 4). In addition, tumor size had a significant impact on OS from uni-covariate Cox proportional hazards model (data not shown). In the multivariable Cox regression analysis, blood transfusion was still significantly associated with worse OS, p = 0.0002, harzard ratio (HR): 2.04 (95% CI: 1.41-2.97)) with the adjustment of age, gender, BMI, ASA, cancer stage, and hemoglobin level in the model (Table 5). In the analysis with the matched cohort, the effect of blood transfusion on OS was still significant (p = 0.035) (Table 5, Figure 2).

**Table 4:** Overall survival (OS) by patient characteristics

**Variable**	**Level**	**N**	**Event**	**Median OS time in months (95% CI)**	**OS rate at 3 years (95% CI)**	**OS rate at 5 years (95% CI)**	**P-value**
	**All patients**	**636**	**243**	**82.39 (78.65,NA)**	**0.72 (0.69,0.76)**	**0.62 (0.58,0.66)**	
Age	< 66	291	83	NA (NA,NA)	0.8 (0.76,0.85)	0.71 (0.65,0.76)	< 0.0001
	> = 66	345	160	74.84 (61.17,NA)	0.66 (0.61,0.71)	0.55 (0.5,0.61)	
BMI	< =25	241	111	77.63 (53.78,NA)	0.65 (0.6,0.72)	0.53 (0.47,0.6)	0.0014
	>25	395	132	NA (79.24,NA)	0.77 (0.73,0.81)	0.68 (0.63,0.73)	
Gender	Female	298	90	NA (NA,NA)	0.81 (0.76,0.86)	0.7 (0.65,0.76)	< 0.0001
	Male	338	153	69.32 (59.99,NA)	0.65 (0.6,0.71)	0.55 (0.5,0.61)	
ASA	2	66	18	NA (77.63,NA)	0.8 (0.71,0.91)	0.72 (0.61,0.85)	0.0405
	3	531	203	NA (79.24,NA)	0.72 (0.69,0.76)	0.62 (0.58,0.66)	
	4	38	22	58.2 (34.33,NA)	0.61 (0.47,0.78)	0.5 (0.36,0.69)	
Stage	1	351	104	NA (NA,NA)	0.82 (0.78,0.86)	0.71 (0.66,0.76)	< 0.0001
	2	131	58	77.63 (62.06,NA)	0.69 (0.62,0.78)	0.58 (0.5,0.68)	
	3	152	81	45.99 (31.64,81.7)	0.53 (0.46,0.62)	0.45 (0.37,0.54)	
Preoperative	N	525	188	NA (81.7,NA)	0.75 (0.71,0.79)	0.65 (0.61,0.69)	0.0001
Hb < 12 g/dL	Y	110	55	58.51 (37.42,NA)	0.6 (0.51,0.7)	0.48 (0.39,0.59)	
Perioperative	N	576	207	NA (79.24,NA)	0.75 (0.71,0.79)	0.65 (0.61,0.69)	< 0.0001
BT	Y	60	36	33.71 (22.5,NA)	0.48 (0.37,0.63)	0.35 (0.24,0.5)	
Number of units	0	576	207	NA (79.24,NA)	0.75 (0.71,0.79)	0.65 (0.61,0.69)	< 0.0001
Transfused	1-3	36	23	30.39 (19.84,NA)	0.47 (0.33,0.68)	0.32 (0.19,0.53)	
	4-9	15	8	54.99 (24.24,NA)	0.56 (0.35,0.9)	0.4 (0.21,0.78)	
	>10	7	4	23.26 (4.76,NA)	0.36 (0.12,1)	0.36 (0.12,1)	
Preoperative	N	532	187	NA (81.7,NA)	0.75 (0.72,0.79)	0.65 (0.61,0.7)	< 0.0001
Chemo-radiation	Y	104	56	51.41 (34.03,NA)	0.58 (0.49,0.68)	0.46 (0.37,0.57)	
Postoperative	Chemo only	137	40	NA (81.8,NA)	0.8 (0.73,0.87)	0.72 (0.65,0.81)	< 0.0001
Chemo-radiation	Chemo-radiation	55	32	50.85 (34.03,NA)	0.58 (0.46,0.73)	0.43 (0.31,0.6)	
	None	408	147	NA (75.53,NA)	0.74 (0.7,0.78)	0.64 (0.59,0.69)	
	Radiation only	35	24	37.52 (23.26,65.05)	0.5 (0.36,0.7)	0.32 (0.2,0.53)	

P-values from the log-rank test (univariable analysis), p < 0.05 is considered statistically significant. *BMI* Body mass index. *ASA* American Society of Anesthesiologist physical status. *Hb* Hemoglobin.

**Table 5:** Association between blood transfusion (Y vs. N) and overall survival

	**p-value**	**HR**	**95% CI**	
Unadjusted (univariate analysis), before matching (E/N = 243/636)	< 0.0001	2.33	1.63	3.32
Adjusted for covariates (multivariate analysis)* (E/N = 243/632)	0.0002	2.04	1.40	2.97
Propensity-matched, stratifying on the matched pair (E/N = 68/135)	0.035	1.83	1.04	3.24

*Covariate in the final model included age, gender, BMI, ASA, Stage, and Hb.

## Discussion

In colorectal cancer patients, a metaanalysis by Amato and Pescatori demonstrated that blood transfusions are an independent factor for cancer recurrence [12]. In our work, we found that perioperative blood transfusions in the context of NSCLC surgery are associated with shorter OS; however, this association was not observed for RFS. Our findings are not fully in line with some of the results published by other authors [2,14,15,18-20].

Pena et al. demonstrated that perioperative blood transfusions did not increase the risk of cancer recurrence or worsen overall survival after lung cancer resection [21]. Similarly, Berardi et al., and Panagopoulos et al. reported that perioperative blood transfusions were not an independent risk factor for poor oncological outcomes [22,23]. Finally, a retrospective study that included 493 patients also found no association between blood transfusions and overall survival after NSCLC cancer surgery [24]. Contrary to those studies and similar to our findings in terms of OS, 5 retrospectives analysis have shown that blood transfusion are associated with shorter OS [2,13-15,25]. Importantly, a recent manuscript published by Churchhouse et al. summarizes the current evidence on this topic and highlights the controversy between blood transfusion and risk of cancer recurrence after lung cancer surgery [4].

There are several reasons that might explain these controversial findings. First, the presence of unknown confounding factors. Second, the indications for blood transfusions and the type of lung resection; in particular the latter (degree and complexity of surgical procedure) has been shown to have a significant impact on recurrence-free survival [26]. Third, it has been suggested that the use of blood transfusions as a surrogate for correction of low hemoglobin values (anemia) may have contributed to the differences amongst the published studies on this topic. It may very well be that preoperative anemia and the myelo-recovery from the disease process and therapeutic interventions may in fact be the more important and determining prognostic factor [22]. And fourth, the statistical methodology used to analysis the data. In this regards, our study is particularly different to the rest of the published studies because we performed a propensity score matching analysis and we consider this a strength of the present work. Propensity matching score analysis has become the suggested method to analyze observational data, in particular in the context of perioperative medicine, because the technique allows estimating the treatment effect controlling for covariates such as comorbidities. In our study, by matching on the propensity score, we eliminated those transfused patients with no comparable non-transfused subjects, hence eliminating potential bias in factors contributing to the decision for perioperative transfusion [27]. In our study, the standardized differences for all covariates were lower than 15% in the post-matching cohort, suggesting substantial reduction of bias between the two groups.

Our univariate analysis also demonstrated that preoperative Hb was associated with poor RFS and OS. It has been suggested that the degree of anemia is a marker of myelosuppression in patients receiving chemoradiation, and in fact, the severity of anemia may be correlated with tumor response and hence an important predictor of survival [28]. Paradoxically, low hemoglobin concentrations may cause tumoral hypoxia which can trigger adaptive mechanisms that may alter the phenotype of the cancer cells turning them more aggressive [29]. Several studies, even those that did not find an association between blood transfusion and NSCLC recurrence, have observed that preoperative anemia is a risk factor for recurrence and it has been hypothesized that blood transfusions may only represent an intervention to correct low hemoglobin concentrations [22,23]. In fact, patients whose hemoglobin levels remained lower than 12 g/dl despite transfusion had worse prognosis, thus suggesting that the degree of anemia before transfusions, may be a predictor of survival [22]. This concept is supported by a study suggesting that after adjusting for preoperative anemia, blood transfusion is an independent factor for recurrence and overall survival in patients with NSCLC stage 1 cancer [13]. In our study, transfused patients had significantly lower preoperative Hb concentration; hence we decided to include this variable in the matching in order to avoid significant confounding.

Another interesting finding of our study is that the number of blood transfusions received appeared to be associated with poor outcomes. There are several possible reasons that might explain our findings. First, a “dose-dependent” effect; although a our findings are not in line with those reported by Keller et al., it is possible to speculate that the magnitude of immunosuppression associated with blood transfusions is greater as the number of units transfused increases; hence, the likelihood to recur after surgery [30]. Second, patients with larger tumors and complex procedures who are subject to a higher chances of incomplete tumor resections and postoperative complications may be also exposed to a higher number of units transfused, hence, our findings only a reflection of the magnitude of bulk tumoral disease [13].

Our study has several limitations. First, this is a retrospective study, hence there are unknown factors that may affect the studied outcomes and were not captured in our data collection (i.e. tumor markers, age of the transfused units, postoperative complications and cause of death). Postoperative complications after major thoracic surgery are known to negatively impact mortality, hence, it is possible to speculate that in our database those patients transfused might have suffered from more transfusion-related or unrelated postoperative complications which would have significantly affected their survival [31]. We were not able to capture the cause of death of all our patients, hence our results might have been heavily confounded by the presence of comorbidities known to have significant impact on OS. In fact, our univariate and multivariate analysis indicated a potential association between ASA physical status and OS; hence, since co-morbidities are part of the ASA score, we included it in the matching analysis.

Second, we included a heterogeneous group of patients in regards to the stage of the disease. Four other studies that analyzed recurrence and mortality in an uniform group of patients (stage 1) have shown that blood transfusions are in fact an independent factor for poor outcomes [2,18,23,25]. Interestingly, Ng et al. suggested that the impact of blood transfusion is less evident in patients with advanced stages of NSCLC because the poor baseline prognosis of this patients [13]. Third, a major limitation of the present work is that we included patients who had received both leucoreduced and non-leucoreduced pRBCs during the perioperative period. The impact of leukoreduced transfusions on NSCLC recurrence and patient survival is controversial. Ng et al. demonstrated that the administration of leukodepleted pRBCs was associated with poorer outcomes than non-transfused patients [13]. Similar results were shown by Panagopoulos et al. suggesting that at least for NSCLC transfusion of pRBCs itself rather than the leukoreduced status of the unit has a more significant impact on outcomes [23]. Blood units given intraoperatively in our institution are typically non-leucoreduced, while the units dispensed to the patient floors are non-leukoreduced. Thus a given patient with multiple perioperative blood transfused may have received leucoreduced units pre- or postoperatively and non-leucoreduced blood intraoperatively. We have therefore not analyzed patients according to the leucoreduction status of the blood units received, as it would not have reflected the current transfusion practice at our institution. Finally, our conclusion of no significant effect of pRBC transfusions on RFS after propensity matching should be studied further in a larger sample size since the Cox regression analysis showed an association between blood transfusion and increased cancer recurrence. It is possible that the small sample size in the matching cohort group may have contributed to our results.

## Conclusions

The perioperative administration of pRBCs appears not to be associated with shorter disease-free survival but with worse overall survival after NSCLC surgery. A randomized controlled trial is needed to support our findings and to elucidate the real impact of perioperative blood transfusion on cancer recurrence and survival after lung cancer surgery.

## Competing interests

The authors declare that they have no competing interests.

## Authors’ contributions

JPC conceived of the study, and participated in its design and coordination and helped to draft the manuscript. VC and HW participated in data collection. LF participated in study design, data analysis and drafted the manuscript. VG participated in study design and drafted manuscript. FM participated in study design and drafted manuscript. AAV participated in study design and drafted manuscript. All authors read and approved the final manuscript.

## Pre-publication history

The pre-publication history for this paper can be accessed here:

http://www.biomedcentral.com/1471-2253/13/42/prepub
